# Hydatid cyst of the foot: a case report

**DOI:** 10.1186/s13256-019-2337-8

**Published:** 2020-01-10

**Authors:** Biniam Ewnte

**Affiliations:** Djibouti Medical Center, Debre Tabor, Ethiopia

**Keywords:** Hydatid cyst, Hydatid of foot, Echinococcosis, Soft tissue hydatid

## Abstract

**Background:**

Hydatid cyst is rarely located in soft tissues. This case of a hydatid cyst over the plantar surface of our patient’s foot is one of the rarest presentations.

**Case presentation:**

This is a case report of a 22-year-old Somali who presented with a lump over the plantar surface of his foot of 1-year duration. The diagnosis of hydatid cyst was made intraoperatively from the typical appearance of a hydatid cyst. The cyst was completely excised. No local recurrence has been detected to date.

**Conclusions:**

The rare location and uncommon incidence made the initial diagnosis of hydatid cyst of the foot difficult. Detection of the typical germinal membrane can guide the diagnosis of hydatid cyst in rare locations that are found incidentally.

## Background

Hydatid disease is due to infection by the tapeworm *Echinococcus granulosus* in its larval or cyst stage. The tapeworm lives in canids, which are infected by eating the viscera of sheep that contain hydatid cysts. It is a significant public health problem in South and Central America, the Middle East, some sub-Saharan African countries, and China. The dog as a pet is the commonest source of infection transmitted to the intermediate hosts: humans, sheep, and cattle. Of all cases of hydatid cyst, 70% of them are formed in the liver. A few of the ova pass through the liver and are caught in the pulmonary capillary bed; ova that escape the pulmonary capillary bed enter systemic circulation, forming cysts in the lung, spleen, brain, or bones. The natural course of the infection varies. Some cysts spontaneously collapse and may disappear or calcify, while other cysts steadily increase in size, displace or compress healthy tissue and organs, and may become complicated. The annual growth rate of the cyst is usually approximately 1–3 cm in diameter [1].

This case report presents a rare location of a hydatid cyst: subcutaneously over the foot. Because such a presentation is very unusual, this case report helps diversify the differential diagnosis of soft tissue masses in such a location, particularly in endemic areas. It also describes in depth the presentation of hydatid disease in such locations. As the finding was incidental intraoperatively, it proposes a mode of treatment in such scenarios.

## Case presentation

A 22-year-old Somali presented to the surgical referral clinic of Djibouti Medical Center (a private hospital located in Hargeisa, Republic of Somaliland) in March 2019 with the main complaint of progressive swelling associated with pain over the left plantar side of his foot. He had no abdominal pain or discomfort, no chest pain or cough. He noticed the swelling 1 year prior to the presentation. There was no history of trauma to the site, no difficulties of walking, no discharge from the swelling, and no lesions noticed on other sites.

He is a college student who lives with his parents; he had no prior medical problems. He has two brothers and a sister. There are no medical illnesses that run in the family. There was no history of tobacco smoking or substance abuse. He has never consumed alcohol.

At presentation, his blood pressure was 120/70 mmHg, pulse rate was 74 beats per minute (bpm), respiratory rate was 18 per minute, and temperature was 36.0 °C axillary. A physical examination of our patient was normal in the rest of his systems. He also had a normal neurological finding. The pertinent finding was an oval-shaped, 2.5 cm wide in diameter, well-circumscribed lump with no increased vascularity, which was non-pulsatile and located over the mid-plantar area of his left foot. It was minimally tender to touch, fluctuant, and with no color change of the overlying skin.

A complete blood count of our patient showed: white blood cells (WBC) 5000 mcL, red blood cells (RBC) 4.7 mcL, hemoglobin (Hgb) 15 gm/dL, hematocrit (Hct) 45%, mean corpuscular volume (MCV) 83, platelets 370 × 10^3^, creatinine 0.7, blood urea nitrogen (BUN) 17, alanine aminotransferase (ALT) 30, aspartate aminotransferase (AST) 28, alkaline phosphatase (ALP) 58, albumin 4.5, total bilirubin 1.2, and direct bilirubin 0.3.

No radiological scan or blood culture was done for our patient. With the initial impression of callus; he was prepared for a minor surgical operation to remove the mass.

## Management and outcomes

Following the initial impression and getting informed consent, he was taken to the minor operation theater. His foot and lower leg were cleaned and draped.

**Fig. 1:**
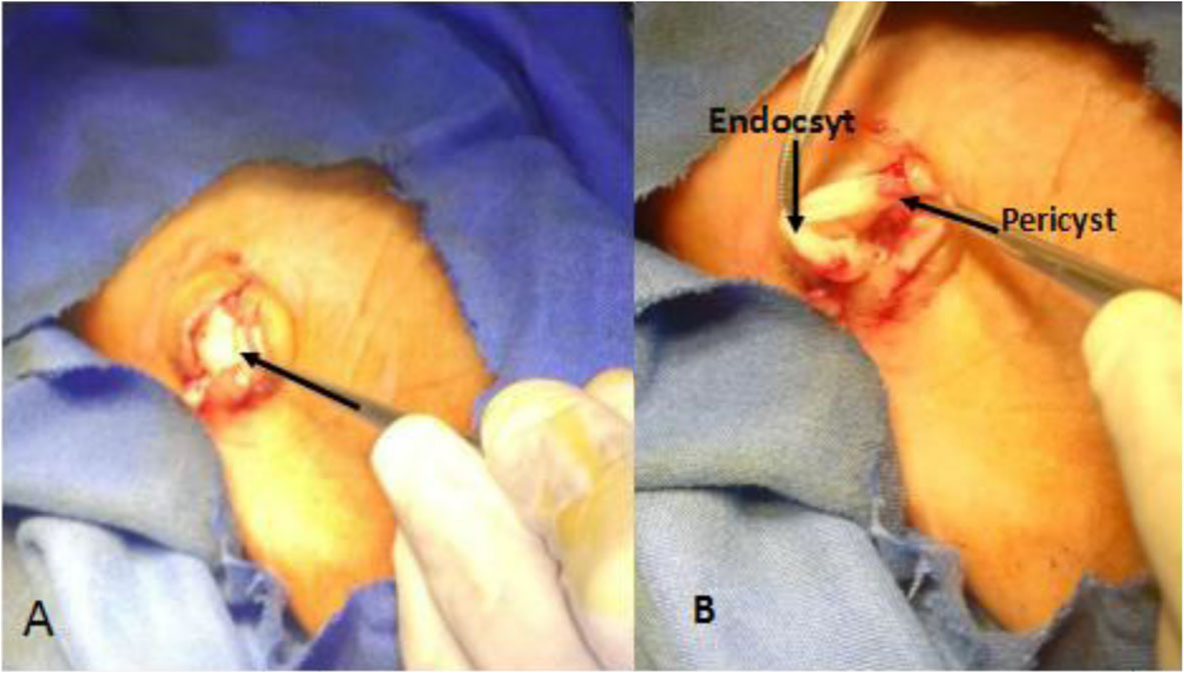
**a** Visible subcutaneous endocyst membrane. **b** Dissected pericyst and endocyst

**Fig. 2:**
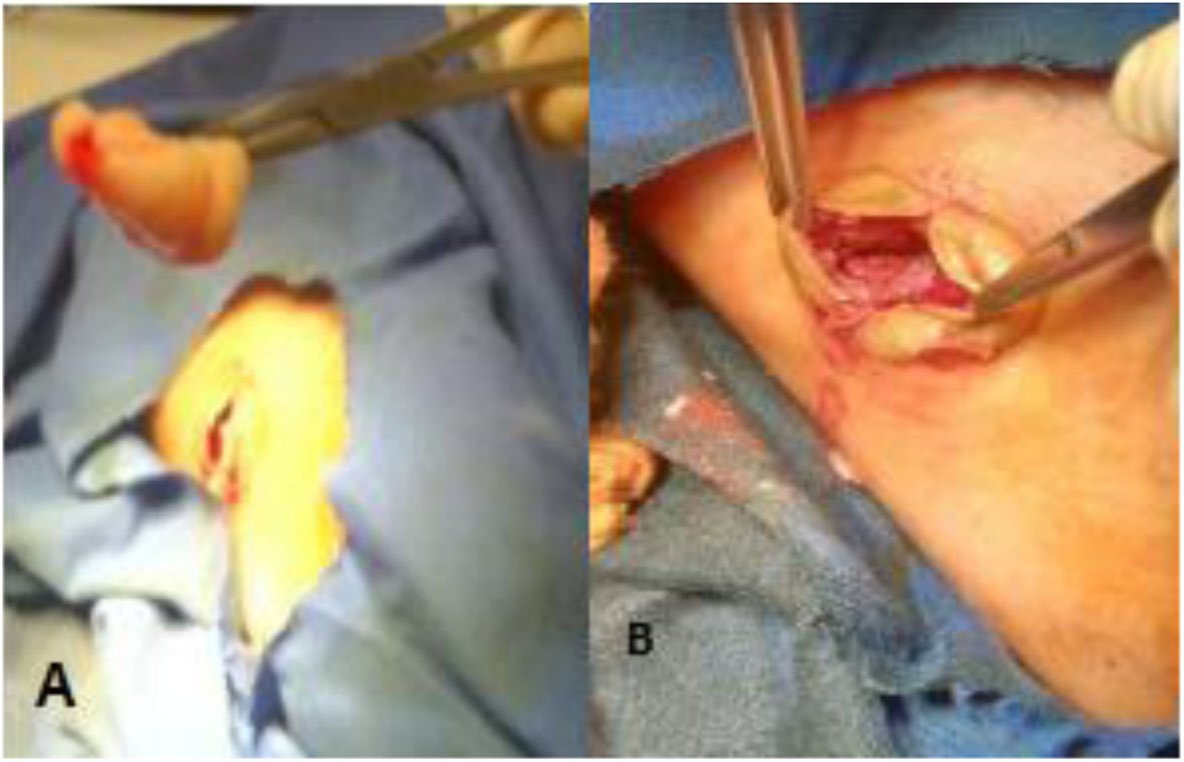
**a** Excised endocyst. **b** Pericyst-lined cavity, post cyst removal

The area of the lump was infiltrated with local anesthesia: lidocaine without adrenaline was used. A transverse incision was made over the lump, followed by dissection of the subcutaneous tissue. Upon exposing the subcutaneous tissue, a typical endocyst membrane covering the hydatid cyst was encountered (Fig. 1). Further dissection revealed the pericyst and endocyst very clearly (Fig. 2). Protecting the surrounding area with iodine-soaked gauze, the endocytic membrane was aspirated, revealing a sandy content.

It was injected with hydrogen peroxide and re-aspirated. Later, the whole intact membrane was dissected out; a pericystic cavity lavage was done using hydrogen peroxide too. The subcutaneous tissue layers and the skin were closed in the standard manner. The specimen was sent for pathology. The pathology result reported a germinative layer which consisted of daughter cysts and brood capsules with scolices and clear fluid content.

Following surgery, he was sent home with diclofenac 50 mg twice a day for 5 days. Follow-up was done a week later, the wound looked ok. The stitches were removed on the tenth postoperative day. Albendazole 400 mg per day was given for 2 weeks’ duration. Subsequently, an ultrasound of his liver to look for possible primary cysts was done and it did not show any focus of a cyst.

He is on follow-up every month at a surgical referral clinic. An abdominal ultrasound was done on the second and fourth month to look for cysts. No local or systemic recurrence of the cysts has been detected so far over the course of 6 months.

## Discussion

This case report presents a rare location of a hydatid cyst which was diagnosed intraoperatively and treated surgically. A subcutaneous location of a hydatid cyst is a rare presentation. The unusual presentation of the case makes the diagnosis difficult prior to an operation. This case report tries to describe the management options of an intraoperatively discovered hydatid cyst. An echinococcal cyst can be located almost anywhere in the body and this should be kept in mind in dealing with a cystic lesion in endemic countries [2]; as this case was found in a non-endemic area it made the preoperative diagnosis challenging.

The main hosts for *E. granulosus* are canids such as dogs, wolves, and foxes, while intermediate hosts include sheep, goats, and cattle. Humans are a coincidental intermediate host. The disease is more frequent in the Middle East, Central Europe, Australia, and South America, where the intermediate hosts are common. A study conducted to estimate the prevalence of hydatid disease in nomadic pastoralists living in eastern Africa showed that the disease was not common among Somalis [3]. The described case is from Hargeisa, Somaliland, which is located in East Africa. Sheep raising is quite common in the area but having dogs as a pet is not common.

Extrahepatic extra pulmonary hydatid cysts were found in 13% of patients with hydatid disease [4]. Hydatid disease presenting in the soft tissues occurs in 0.5–4.7% of patients.

Preoperative diagnosis of a hydatid cyst can be challenging and can sometimes be misdiagnosed as an abscess [5, 6]. The initial diagnosis of the case was a dermoid cyst as the area is not an endemic area and the site was a very rare location for a hydatid cyst.

A palpable mass is the most constant clinical or exploratory finding of hydatid disease affecting soft tissues [7]. Our patient presented with pain around the mass which could be explained by mass effect.

A hydatid cyst can present as isolated subcutaneously without involving other organs; a rare report of such an incident involving the face was reported by Öztürk *et al*. [8]. Subcutaneous hydatid cyst may be secondary or primary. In secondary cysts, there is a primary location of hydatid disease such as liver, lung, or spleen that is operated on or not operated on [9].

Isolated subcutaneous involvement of hydatid over the thigh, palms, and calf with no other organ involvement has also been reported [10, 11]. Salamone *et al.* reported that 45% of soft tissue hydatid cysts were located in the lower limb [12]. Also, reports of hydatid cyst in the foot, which affected the cuneiforms, and the navicular and talus bones have been published [13, 14]. A case of a hydatid cyst with a subcutaneous location of the foot has not been found in searches.

Operative spillage of cyst contents can lead to local regrowth of hydatid cysts after a suitable interval [15]. Complete excision of the germinal layer following injection of sporicidal agents, preventing the spread of germinal cyst to the surrounding tissue, is the commonly practiced mode of treatment. Hydrogen peroxide is one of the suggested sporicidal agents [16].

Treatment with albendazole in *E. granulosus* infection can result in an apparent cure in up to 30% of cases. Duration of therapy and dose are also important, and with albendazole, efficacy seems to increase with exposure up to 3 months in the commoner cyst sites. The issue of cyclical versus continuous treatment still has to be resolved [17]. As our patient had a very rare site for a hydatid cyst and there was no intraoperative spillage, he was treated for only 2 weeks with albendazole. He has been on follow-up for the past 6 months, and there has been no recurrence noted so far.

The rarity of this case made preoperative diagnosis difficult because our area is not an endemic one and the location of the cyst was very unusual. Intraoperative diagnosis of the typical germinal cyst membrane, with complete excision, was effective for an incidentally discovered subcutaneous hydatid cyst on the plantar side of our patient’s foot.

## Conclusion

Although hydatid cyst is rare, a high index of suspicion and identification of the typical endomembrane of the cyst could guide intraoperative diagnosis. Chemotherapy with albendazole for a short duration of 15 days could suffice for a subcutaneously located hydatid cyst if the cyst is removed intact with no local spillage. So far, for our patient, no local or systemic recurrence has been detected.

## Data Availability

All the images and detailed information of this case are available at Djibouti Medical Center digital patient record and can be submitted upon request.
